# Emerging Nutrition Approaches to Support the Mind and Muscle for Healthy Aging

**DOI:** 10.21926/rpn.2204022

**Published:** 2022-11-16

**Authors:** Gabrielle M Mey, Jacob T Mey

**Affiliations:** 1 Lerner Research Institute, Department of Neurosciences, Cleveland Clinic, Cleveland, OH, 44195 USA; 2 Pennington Biomedical Research Center, Baton Rouge, LA, 70808 USA

**Keywords:** Medical nutrition therapy, ketogenic, MIND, calorie restriction, muscle protein synthesis

## Abstract

This narrative review highlights recent advances and ongoing trials using nutrition approaches for healthy aging. Focus will be placed on nutrition therapies that target cognition (“the mind”) and mobility (“the muscle”), both critical components to maintaining a high quality of life for older adults. For “the mind,” two seemingly incongruent therapies are being investigated to improve cognition–the MIND diet (high in carbohydrates and anti-oxidant fruits and vegetables) and the ketogenic diet (low in carbohydrates, high in fats). For “the muscle,” a focus on protein and energy intake has dominated the literature, yet a recent clinical trial supports the use of whole-grains as a tool to improve whole-body protein turnover–a primary regulator of lean body mass and muscle. Finally, emerging data and clinical trials on caloric restriction have solidified this strategy as the only nutritional approach to slow intrinsic factors of whole-body aging, which may positively impact both “the mind” and “the muscle.”

## 1. Introduction

The world population is rapidly approaching a new era, where older adults (age 65+ years) outnumber children. This unprecedented global demographic shift is anticipated to occur by 2034 [1]. With an ever-growing aging population, the health needs of older adults are at the forefront of research interest. Aging is an inevitable and multifactorial process that culminates in a progressive loss of physiological function [2]. The aging process presents with impairments in many critical functions; here, we focus on two critical functions that contribute to the loss of independence, reduction of quality of life [3] and increased mortality risk [4]: cognition (“the mind”) and physical function (“the muscle”). Loss of cognition ranges from short-term memory deficits to advanced dementia, processes which may be accelerated by sub-optimal nutrition intake [5]. Similarly, muscle mass declines at about 8% per decade after age 40 years and accelerates to ~15% per decade after age 70 years resulting in profound loss of both muscle mass and strength [6], again accelerated by sub-optimal nutrition intake [7]. Both cognition and muscle function are hallmarks of healthy aging, and maintaining these core functions can improve the independence and quality of life of the later years of life (i.e., increase healthspan) [8]. This mini-review will focus on the emerging nutritional approaches that have potential to prevent or reverse the decline in cognition (“the mind”) and physical function (“the muscle”) that occur with aging in older adults. Importantly, this mini-review aligns with the World Health Organization’s (WHO) commitment to a global strategy on healthy aging. This emphasizes current evidence for and future use of nutrition recommendations to support the functional ability of an aging population to not only meet basic needs, but thrive and continue to contribute to society [9].

The core population discussed in this mini-review are older adults that are otherwise healthy. Critically, common noncommunicable diseases such as cardiovascular disease, diabetes and obesity will dramatically affect nutrition recommendations. As such, medical nutrition therapy is established for these diseases to provide focused food and supplement guidelines. However, in healthy individuals where the primary concerns are functions of the natural aging process, medical nutrition therapy is not established. Therefore, this review targets emerging approaches that may benefit the process of aging *per se*, focusing on cognitive and physical function. It is noteworthy that micronutrient status may be impacted in older adults (e.g., Vitamin B12, Vitamin D), and may differ on both a population and individual basis; micronutrient bioavailability and micronutrient status is beyond the scope of this review which focuses on dietary patterns, macronutrient composition and total calorie intake. To form our opinions, we compiled information from clinical trials and population studies ranging across the globe. As such, readers should be cognizant to differences in local cuisine, food availability and food culture when considering the nutrition information in this review for specific populations. Of particular concern in older adults is food insecurity, which may make implementation of some of these nutritional approaches difficult. With these considerations, this review offers insight into the developing nutritional approaches relevant to a healthy aging population.

## 2. The Mind

Cognitive function plays a critical role in normal daily function and is imperative to maintaining health and independence throughout the aging process. Components of cognitive function include attention, memory, verbal fluency, abstract reasoning and visuospatial skills [10]. Loss of cognitive function is not only associated with reduced quality of life, but also increases the risk for development of Alzheimer’s disease [11] or other dementias [12]. Therefore, maintaining cognitive function throughout aging is of utmost importance for the health and wellness of older adults. Emerging evidence suggests two distinct nutrition therapies may support cognitive function: the MIND diet and the ketogenic diet.

### 2.1 The Mind Diet

The MIND diet was developed by Dr. Martha Clare Morris and colleagues out of Rush University Medical Center in Chicago, IL [13]. It incorporates neuroprotective dietary components from two diets associated with better cognitive function in older adults [14]: the Mediterranean and DASH (Dietary Approaches to Stop Hypertension) diets [15]. Thus, the MIND diet acronym stands for **M**editerranean-DASH Diet **I**ntervention for **N**eurodegenerative **D**elay. The diet was developed from the data of a unique longitudinal population study, the Chicago Health and Aging Project [16]. The MIND diet places a focus on plant-based foods, fish and poultry, while minimizing saturated fats and added sugars. Similar to other healthy dietary patterns, it is high in carbohydrates and moderate in proteins and fats. A typical MIND diet would include daily green leafy vegetables and whole-grains along with protein foods such as fish and poultry. Unique to the MIND diet is an additional emphasis on berries, recommending 2 or more servings per week, nuts (5 or more servings per week), and beans (4 or more servings per week). Other foods are recommended to be consumed sparingly, which include high saturated fat or high sugar items, such as red meats, butter, cheese, refined grains, and fried foods.

Epidemiological studies have identified that several dietary patterns are associated with protection from cognitive decline or dementia, including the DASH and Mediterranean diets [17, 18]. One critical study that followed these large-scale associations was conducted by Morris et al. as part of the Rush Memory and Aging Project, which investigated whether the MIND diet was protective against cognitive decline or dementia. This study recruited over 1,500 volunteers from retirement communities and senior public housing and followed them for 4.5 years on average. Participants underwent annual neurological exams, which included cognitive tests and assessment for Alzheimer’s disease, and reported their diets through food frequency questionnaires. This study showed that adherence to either the DASH, Mediterranean, or MIND diets provides cognitive protection. Importantly, only a modest adherence to the MIND diet may provide significant cognitive protection, whereas a high adherence was required for either the DASH or Mediterranean diets to confer the same protection [19]. Since its development, many trials have been conducted on the MIND diet and its role in cognitive health. A recent systemic review corroborated the benefits of the MIND diet on cognition, reporting that the MIND diet was positively associated with cognition and global cognitive function, specifically in older adults [20]. It further reported that the MIND diet outperformed other diets such as the DASH, Mediterranean and other plant-based diets. Taken together, the MIND diet is consistently associated with cognitive health and in older adults may be superior to other similar dietary patterns.

The biological mechanisms underlying cognitive decline and Alzheimer’s disease remain unclear, and therefore effective pharmacologic interventions are lacking [21]. While the link between the MIND diet and improved cognitive health is not fully understood, information from pre-clinical studies, epidemiologic associations and our understanding of human physiology have led to hypotheses for nutrition-related mechanisms. Primary nutrition factors include antioxidants, B vitamins and omega-3 fatty acids (reviewed in [22]). Oxidative stress is a pillar of physiologic aging [23] and is implicated in neurodegeneration, such as seen with cognitive impairment and Alzheimer’s disease [24]. Antioxidants neutralize free radicals that cause oxidative stress. Nutrients that are antioxidants include vitamins A, C, and E and phytochemicals such as flavonoids or carotenoids. Foods that have high amounts of A, C, E or related phytochemicals include colorful vegetables and fruits as well as nuts – foods that are consistent with the MIND diet. The B vitamins are not antioxidants, but play unique roles in neurocognitive function. They are critical for neural development (folate, B9, prevents neural tube defects during pregnancy) while deficiency of B12 in adults includes neurological symptoms like peripheral neuropathy and cognitive impairment. B12 deficiency is common in older adults due to either increased food intake or a reduced ability to absorb B12 from foods. Notably, a recent systematic review suggests that B vitamin supplementation may delay cognitive decline in older adults [25]. Additionally, elevated plasma homocysteine (which reflects the functional status of the B vitamins folate, B6 and B12) is associated with dementia and cognitive decline [26–28], while the MIND diet has just recently been shown to lower plasma homocysteine [29]. Foods that have high amounts of B vitamins include fish, poultry, green leafy vegetables and legumes – consistent with the MIND diet. Finally, omega-3 fatty acids are polyunsaturated fatty acids that must be obtained from the diet. Omega-3 fatty acids (particularly docosahexanoic acid or DHA) are present in the brain. Omega-3 supplementation in animal models has been shown to aid in neural development and emerging data in humans suggest improved cognitive function in adults [30]. Foods that have high amounts of omega-3 fatty acids are fish and nuts – consistent with the MIND diet. Ultimately, the MIND diet shows potential for preserving cognitive function through the aging process, but interestingly, a distinct low-carbohydrate approach is now seeing parallel interest for preserving cognitive function in aging.

### 2.2 The Ketogenic Diet

The classic ketogenic diet utilizes a low-carbohydrate and high-fat macronutrient profile, which causes elevated production and oxidation of ketones. In contrast to the recently developed MIND diet, the ketogenic diet was first used clinically to treat epilepsy in the 1920s [31, 32]. The brain is a highly metabolic organ, accounting for about 20% of the body’s basal metabolic rate [33]. The brain relies primarily on glucose to provide its energy needs, except when glucose availability is scarce (as is the case on a low-carbohydrate, ketogenic diet or with prolonged fasting), at which point ketones become the predominant energy source for the brain [34]. Common foods on the ketogenic diet include foods high in fat, such as red meats, cheeses, or full-fat dairy. While many high-fat foods are not consistent with the MIND diet, some foods do overlap well including nuts and fatty fish, like salmon. Other ketogenic food staples include non-starchy vegetables, like leafy greens. Aside from food staples, restricted nutritional components are just as important to maintaining an effective ketogenic diet. To achieve a low-carbohydrate diet, consumption of carbohydrates typically falls between 20–50 grams per day. This requires an elimination of high-carbohydrate foods such as starchy vegetables like potatoes, legumes, grains, cereals and juices. Finally, in contrast to the MIND diet, ketogenic diets utilize butter for cooking, which is high in saturated fats, whereas the MIND diet would utilize oils with poly-and monounsaturated fats such as olive oil. Despite these distinct differences in dietary profiles, emerging evidence suggests the ketogenic diet, similar to the MIND diet, may improve cognition with aging and prevent age-related neurodegenerative disorders.

The restrictive ketogenic dietary pattern, unlike the MIND diet, is not widely followed and therefore epidemiologic associations between the ketogenic diet and healthy brain aging are limited. However, several short-term clinical trials offer insight into the effect of a ketogenic diet on “the mind.” Krikorian et al. randomized 23 older adults to either a ketogenic diet or a high-carbohydrate control diet for 6 weeks [35]. All adults had mild cognitive impairment (a risk factor for Alzheimer’s disease) with an average age of ~70 years and were largely college educated (average education years >15 years). After a 6-week ketogenic diet, participants improved verbal memory performance compared to the high-carbohydrate control diet. Further, the concentrations of blood ketone levels positively correlated with memory performance. In a similar study, Brandt et al. randomized older adults with mild cognitive impairment to a 12-week ketogenic or high-carbohydrate control diet [36]. Although only 14 participants completed the trial, with modest dietary adherence, individuals adhering to a ketogenic diet improved episodic memory scores and positive mood states. Another small trial showed the ketogenic diet improved a cognitive component of the Alzheimer’s disease Assessment scale over a 12-week intervention [37]. Finally, yet another small trial conducted by Sheffler et al. included 9 older adults who were counseled to consume a ketogenic diet for 6 weeks, after which they observed significant improvement in cognitive performance [38]. Taken together, these small clinical trials support the ketogenic diet as a potential therapeutic strategy for mild cognitive impairment with aging. Certainly, larger clinical trials are warranted to better understand this therapeutic potential, while the underlying mechanisms continue to be more fully elucidated.

The biological mechanisms linking a ketogenic diet and cognitive health are primarily thought to be related to nutrient metabolism (i.e., the transition from utilizing carbohydrates to utilizing more fats and ketones). The development of cognitive impairment and Alzheimer’s disease are related to impaired glucose metabolism in the brain (hypometabolism of glucose) [39]. This glucose hypometabolism in the brain occurs with normal aging, is accelerated in the cognitively impaired, and is postulated to be due to reduced glucose transport [40]. Since the landmark findings of Owen et al. in 1967 showing that ketones replace glucose utilization in the brain when glucose availability is lacking [41], there has been a rising interest in the impact of shifting brain nutrient utilization. Additional information can be gained from studies that experimentally increased circulating ketones using medium chain triglycerides (MCT). Six months of MCT supplementation increased circulating ketones, uptake of ketones into brain white matter, and processing speed from a neurocognitive battery test [42]. A systematic review further showed that MCTs increase ketones and may improve cognitive impairment. Other proposed mechanisms for the neuroprotective potential of the ketogenic diet include preservation of mitochondrial function [43], protection against neuroinflammation [44] or structural changes [45]. However, studies on MCTs and the ketogenic diet remain inconsistent and have additional risk of bias [46] (e.g., lack of pre-specified analysis plans, adequate controls, adequate blinding). To address these current limitations, in particular the high risk of bias, more rigorous randomized clinical trials with sufficient blinding of both the research participants and research teams (e.g., caregivers, outcomes assessors, biostatisticians) are needed.

### 2.3 Nutrition for “The Mind” Summary

Preserving cognitive function during aging is critical to maintaining independence and quality of life in older adults. Two distinct dietary approaches show promise for preserving cognitive function with aging: the MIND diet and the ketogenic diet. The proposed mechanisms that preserve cognitive function are distinct to each approach, but remain to be fully elucidated. Clinical trials are currently limited by small sample sizes, inconsistent methods, and subsequently incongruent findings, particularly for the ketogenic diet. Inconsistencies in control groups, blinding, and analysis plans introduce a high risk of bias, which hinder the clinical utility of these nutritional approaches. Therefore, no established medical nutrition therapy exists to prevent cognitive decline throughout the aging process. Given the growing need to support an ever-aging population, determining therapeutic nutritional approaches to support “the mind” is of prime research and public health interest.

## 3. The Muscle

In comparison to emerging data on “the mind,” nutrition to support “the muscle” has a more established history, which has led to concrete nutrition recommendations. Still, recent findings have implicated novel nutritional components that may affect “the muscle” and suggest we may have more to learn about the role of nutrition and the muscle throughout the aging process. Skeletal muscles play a critical role in locomotion and performing day-to-day activities. Loss of muscle mass and function with aging contributes to immobility, increased mortality, and reduced quality of life [47]. Although a decrease in muscle mass occurs as a natural part of the aging process [6], excessive loss leads to sarcopenia, a disease that only recently gained a consensus definition [48, 49] and clinical diagnostic coding [50, 51]. Therefore, maintaining muscle mass and function with aging remains a critical part of healthy aging. It is well established that inadequate protein and/or energy intake (i.e., kilocalorie intake) dramatically increases the risk for muscle loss and the development of sarcopenia in aging. Thus, the most effective nutrition recommendations are to optimize protein and energy intake to support disease-free living throughout aging. Indeed, medical nutrition therapy for suboptimal protein and/or energy intake concomitant with sarcopenia includes increasing protein and energy intake through diet or dietary supplements. Despite these nutritional therapies, muscle loss and increased risk of developing sarcopenia remain a concern in aging. Excitingly, emerging data suggest novel nutritional approaches beyond these established recommendations may be necessary to optimally maintain muscle mass and function with aging.

### 3.1 Established Recommendations for Protein & Energy Intake

Protein and energy intake are essential for the maintenance of lean body mass and muscle, whereby adequate intake is required for both growth and preservation. Total energy intake to maintain body mass is a necessity, but protein intake *per se* is required for the maintenance of a positive nitrogen balance and preservation of lean body mass, including muscle. National guidelines recommend the consumption of 0.8 grams of protein per kilogram of body weight for adults regardless of adult age [52]. However, a persistently growing body of literature has evidenced higher protein intake as a protective measure to the maintenance of lean body mass and muscle mass throughout aging [53, 54]. The traditional recommendations of 0.8 grams of protein per kilogram of body weight may have been low be due to limitations in the original methods (nitrogen balance) used to develop the recommendations [55], the populations used to develop them [56, 57] or new information on reduced protein synthesis responses to protein intake in older adults [58]. Regardless of the reason for this discrepancy, an international group of experts have developed new recommendations for older adults of 1.0–1.2 grams of protein per kilogram of body weight [59]. Greater protein intake is also consistent with epidemiologic association studies, whereby greater protein intake is related to greater lean body mass [60, 61] and physical function [62, 63].

Regarding the impact of aging on skeletal muscle, a potential biological mechanism underlying the loss of muscle mass over time is an impaired protein synthesis response to protein intake. Skeletal muscle mass is regulated by the net effect of protein synthesis (muscle accretion or growth) and protein breakdown (muscle loss). While basal rates of protein synthesis are similar in young and old adults, rates of protein synthesis in response to protein ingestion (i.e., meals) is blunted in older adults [64]. A maximal protein synthesis response is achievable in older adults if protein intake is increased (e.g., 40 gram protein bolus versus 20 gram protein bolus) [65]. This protein bolus to achieve a maximal protein synthetic response is greater in older adults than in young adults, based upon comparisons between dose-response studies previously conducted in young adults [66]. The requirement to ingest greater protein to elicit a maximal protein synthesis response in older adults is termed “anabolic resistance” [54]. Although optimal total and per-meal protein dose for the preservation of muscle mass with aging remains to be determined, consuming 1.2 grams of protein per kilogram body weight dispersed over 3 or more meals on the backdrop of adequate calorie intake to maintain body weight appears promising.

### 3.2 Emerging Evidence on Whole-Grains

The predominance of the literature regarding nutritional approaches to preserve muscle mass and function in aging is targeted at protein and energy intake – and rightly so. The evidence is profound and clear that protein and energy intake play a dominant role in the regulation of lean body mass, including muscle. Excitingly, emerging evidence implicates whole-grains as a novel nutritional factor to maintain a positive protein balance and potentially preserve muscle mass and function in aging. Whole-grain consumption is a pillar of national nutrition guidelines [67], while epidemiologic association studies show that whole-grain intake improves body composition (relatively more lean mass compared to fat mass) [68–70]. Despite this, few interventional trials on whole-grains and lean body mass or the underlying physiology of protein turnover (synthesis and breakdown) have been conducted and the literature remains inconclusive [71–74]. However, a recent feeding trial has offered new insight into the potential role of whole-grains in lean body mass. Kirwan et al. designed a cross-over feeding trial comparing eight weeks of a whole-grain versus refined-grain diet in middle-aged adults. Diets were matched for macronutrient content and were isocaloric, differing only in the presence of whole-grains or refined-grains (primary grain components included oats, rice, wheat, and barley) [75]. The primary outcomes report did not reveal differences in body composition, although we recently published a secondary outcomes report that included whole-body protein turnover assessed by two stable isotope tracers. This revealed a more positive 24-hour integrated net protein balance on a whole-grain diet, with no difference in net balance when observing the fasted state in isolation [76]. These data suggest whole-grain intake may support a more positive protein balance in combination with adequate protein and calorie intake. Although we cannot derive a potential effect on skeletal muscle from whole-body protein turnover, skeletal muscle comprises ~1/3 of whole-body protein turnover and is the body’s largest mobile reservoir of amino acids/nitrogen. In this report, we conducted an *in vitro* investigation in skeletal muscle cells, showing a whole-grain wheat bran extract increases global protein synthesis rates. Finally, to investigate whether whole-grain intake has an appreciable effect on aging, we analyzed the National Health and Nutrition Examination Survey (NHANES) and showed whole-grain intake was positively associated with greater walking speed in older adults, a representative measure of muscle function. Despite our interesting and provocative report, protein and energy intake remains the primary nutritional effectors of muscle mass and strength with aging. Future research will be required to assess the potential therapeutic effect of adding optimal whole-grain intake on the backdrop of these established nutritional approaches.

### 3.3 Nutrition for “The Muscle” Summary and a Caveat for the Profound Effect of Physical Activity

Adequate protein and energy intake persist as the primary nutritional factors that protect skeletal muscle in aging. Although national guidelines have not adjusted, recent work suggests a protein intake above 1.2 grams of protein per kilogram of bodyweight per day (split among 3 or more meals) supports muscle mass and strength with aging. It is important to note that despite established (protein and energy intake) and emerging (whole-grain) nutrition factors, another major factor is not related to diet at all, but rather to physical activity and resistance exercise training [77]. The effect of physical activity and exercise is so potent, that it even brings into question whether additional protein intake or energy provision provides benefit beyond exercise in isolation (at least in mobility-limited older adults) [78]. Certainly, an integrated approach including both physical activity, resistance exercise training, and optimal nutrition is prudent and supported by the literature. Still, further research is necessary to determine the precise recommendations that can effectively preserve muscle mass and function in aging.

## 4. Calorie Restriction with Optimal Nutrition: A Universal Tool?

It is noteworthy to mention that cognitive and physical function are consistently linked in observational studies [79], and thus addressing one may be critical to addressing the other. Chronic calorie restriction on the backdrop of optimal nutrition (micro- and macronutrient intake) has recently gained interest as an anti-aging tool with the potential to impact both “the mind” and “the muscle” [80]. Calorie restriction with optimal nutrition (henceforth referred to as “calorie restriction”) does not focus on *what* you eat, but rather *how much* you eat. Commonly, we focus on obesity, where weight loss is the goal and provides indisputable benefits to human health. However, calorie restriction even in otherwise healthy adults may bestow unique anti-aging properties that impact both “the mind” and “the muscle.” A plethora of pre-clinical work including drosophila [81, 82], murine models [83, 84] and non-human primates [85–87] have established calorie restriction as the only non-genetic method that increases lifespan. Logistical limitations prevent long-term human trials of longevity (e.g., maximal human lifespan is ~120 years compared to just ~2 months for drosophila, ~2 years for murine models or ~40 years for non-human primates). Still, human observational studies in Blue Zones [88], the Biosphere II experiment [89], and the Calorie Restriction Society International [90] suggest a moderate calorie restriction of ~12% less than habitual intake improves indicators of longevity and population health. Recently, a randomized clinical trial, CALERIE II showed a similar level of calorie restriction improved biological measures of aging (DNA damage) [91] without negative effects on cognitive performance [92] or muscle function [93]. Of note, both body weight and muscle mass were reduced after calorie restriction and the potential risks of aging with a lower body weight and muscle mass such as sarcopenia, frailty, and immobility remain to be empirically determined. Taken together, calorie restriction in the context of optimal nutrition may be a universal tool to slow the aging process. Given the aging process includes a loss of both muscle and cognitive function, a universal slowing of the aging process may aid in the preservation of both “the mind” and “the muscle.”

## 5. Conclusion

In a globally aging population, preventing the age-related decline in cognitive and physical health is of prime importance to maintaining the independence and quality of life of the older adult population. Emerging evidence suggests nutritional factors may support both cognitive and physical function with aging (i.e., support “the mind” and “the muscle”). This review has highlighted the recent evidence on nutritional strategies to combat cognitive and physical decline, including the potential underlying biological mechanisms. Two nutritional strategies that may support cognitive function are the MIND and ketogenic diets. The MIND diet may work through phytonutrient and antioxidant mechanisms, while the ketogenic diet may work through shifting nutrient utilization towards ketones. To support physical function, an established strategy is the use of high-protein/energy diets, while early evidence supports the potential for a new strategy, which is increasing the intake of whole-grains. To date, only one nutritional strategy is evidenced to support a universal slowing of the aging process and therefore has potential to support longevity along with preventing the age-related decline in cognitive and physical function: calorie restriction with optimal micronutrient provision. It is notable that despite these recent advances, substantial additional research is required to establish evidence-based nutritional approaches to support “the mind” and “the muscle” throughout the aging process.
